# Suppression of type I collagen in human scleral fibroblasts treated with extremely low-frequency electromagnetic fields

**Published:** 2013-04-12

**Authors:** Jie Wang, Jiefeng Cui, Huang Zhu

**Affiliations:** 1 Department of Ophthalmology, Xinhua Hospital, Shanghai Jiao Tong University School of Medicine, Shanghai, China; 2 Liver Cancer Institute, Zhongshan Hospital, Fudan University, Shanghai, China

## Abstract

**Purpose:**

To investigate the expression differences of type I collagen (*COL1A1*) and its underlying mechanisms in human fetal scleral fibroblasts (HFSFs) that were treated with conditioned medium from retinal pigment epithelial (RPE) cells under extremely low-frequency electromagnetic fields (ELF-EMFs).

**Methods:**

The ELF-EMFs used in this study were established by slidac and artificial coils. Growth of the treated HFSFs was evaluated by a cell-counting kit-8 assay. The expression of *COL1A1* and matrix metalloproteinases-2 (*MMP-2*) in the treated HFSFs was detected by reverse transcription PCR (RT-PCR) and western blot, and the expression of transforming growth factor-β2 (TGF-β2) and basic fibroblast growth factor-2 (FGF-2) in RPE cells exposed to EMFs was detected by RT-PCR. The expression of *COL1A1* and *MMP-2* in HFSFs was further confirmed by immunofluorescence staining. Activation of extracellular signal-regulated kinase 1/2 (ERK1/2 also called p44/p42 mitogen-activated protein kinases [MAPK]) and p38 in HFSFs was measured by western blot.

**Results:**

We found that exposure to ELF-EMFs resulted in a decreased proliferation rate of HFSFs and that addition of RPE supernatant medium could enhance this effect. Compared with that of the control cells, a significant decrease in collagen synthesis was detected in HFSFs under ELF-EMFs. However, the expression of MMP-2 was upregulated, which could be further enhanced via an RPE supernatant additive. The activities of ERK1/2 and p38 were significantly increased in HFSFs exposed to ELF-EMFs, and this effect could be enhanced by RPE supernatant medium additive.

**Conclusions:**

Our results suggested that ELF-EMFs can inhibit the expression of type I collagen in HFSFs and contribute to the remodeling of the sclera.

## Introduction

Side effects of extremely low-frequency electromagnetic fields (ELF-EMFs) on public health, especially on ocular health, have recently attracted more attention than they have received in the past. Morphological alteration of the conjunctiva and reductions in the number of goblet cells could result from 50 Hz ELF-EMFs at 1.5 mT [1]. Several studies have also demonstrated that there are relationships between electromagnetic radiation (EMR) and cataract [2,3], corneal [3,4], retinal damage [4]. However, little is known about the biologic function of scleral changes exposed to ELF-EMFs.

The human sclera is composed mainly of type I collagen fibrils (50% to 70%), which mostly distribute among the equator and posterior pole region of the eyeball [5]. Scleral thinning involves a net loss of matrix, smaller diameter collagen fibrils in the sclera, a net loss of scleral tissue through reduced collagen synthesis, and increased degradation [6,7]. Scleral fibroblasts, which reside between the collagen fiber and bundle lamellae, could monitor changes of the surrounding extracellular matrix [8,9]. The proliferation of scleral fibroblasts changes with the decrease in proteoglycan synthesis, which could affect the biomechanics of sclera and induce elongation of the eye [10]. The remodeling of sclera and the extension of axial length could induce refractive errors, retinal degeneration, and/or detachment [11,12]. In our preliminary experiments, we found that ELF-EMFs could lengthen the axial length of guinea pig’s eyes and induce a structure disorder of collagen fibrils in guinea pig scleral. Information on the biologic effects of ELF-EMFs on scleral fibroblasts, therefore, is of interest and has never been investigated.

It is well known that the retina is the source of ocular growth-regulating signals and that retinal pigment epithelial (RPE) cells are intimately involved in eye growth regulation [13]. Increasing evidence has shown that the retina could synthesize and secrete cytokines and enzymes (such as transforming growth factor-β [TGF-β] and basic fibroblast growth factor [bFGF]) to regulate the remodeling of sclera [14-17]. However, no report has addressed the biologic function of human fetal scleral fibroblasts (HFSFs) exposed to ELF-EMFs or HFSFs treated with RPE supernatant medium.

Matrix metalloproteinases (MMPs) are secreted by different kinds of cells, such as fibroblasts and inflammatory cells [18]. MMPs can degrade one or several components of extraceellular matrix (ECM) and promote ECM remodeling [19]. It is well recognized that high expression of MMP-2 could lead to collagen degradation [20], which plays a crucial role in the regulation of scleral pathological remodeling [21]. The *MMP-2* gene was found to be involved in human refractive variation [22] and participated in altering the extracellular matrix in the posterior sclera [23]. An elevated level of active MMP-2 expression has been detected in induced tree shrews with myopia [24]. Moreover, MMP-2 might be involved in tissue remodeling through activating extracellular signal-regulated kinase 1/2 (ERK1/2, also called p44/p42 mitogen-activated protein kinase [MAPK]) and p38 MAPK [25,26]. EMF exposure could activate multiple downstream signaling pathways, such as ERK signaling [27]. We speculated that ELF-EMFs might upregulate MMP-2 expression and activity by activating ERK1/2 and p38 MAPK signal molecules.

In this study we used an EMF with 0.2 mT intensity, which is the upper safety limit for public exposure to non-ionizing radiation in the guidelines of the International Commission on Non-Ionizing Radiation Protection [28]. Our objective was to detect the effect of ELF-EMFs on sclera by evaluating their influence on proliferation and content of type I collagen in cultures of HFSFs. We then investigated the effect of ELF-EMF exposure with and without RPE supernatant additive on the biologic function of HFSFs and explored the underlying mechanisms of RPE supernatant influence on HFSFs.

## Methods

### Cell culture

HFSFs were obtained from Beijing Institute of Ophthalmology (Beijing, China), and RPE cells (ARPE19) were obtained from the American Type Culture Collection. HFSFs and RPE cells were cultured in Dulbecco’s modified Eagle’s medium (DMEM, Gibco, Carlsbad, CA) with 1% antibiotic/antimycotic (penicillin–streptomycin; Invitrogen) and 10% fetal bovine serum (FBS; Gibco), and incubated at 37 °C in a humidified incubator containing 5% CO_2_. The growth medium was changed every 3 days. When the cultures began to reach 80% confluence, cells were trypsinized for 1 min at 37 °C in 0.25% trypsin/EDTA, and then subcultured at a split ratio of 1:3 in 25-mm^2^ plastic bottles.

### Exposure system and Experiment design

**Figure 1:**
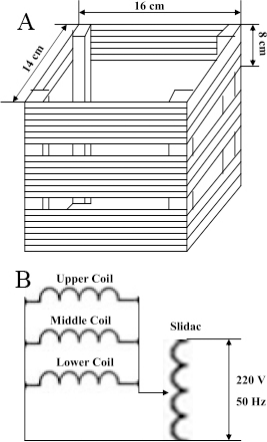
Exposure systems. **A**: ELF-EMFs generating system is composed by three horizontally parallel Helmholtz coils (16 cm length, 14 cm width and 8 cm height) and one slidac. Samples were placed in the space between the coils. **B**: This is a schematic of EMF experimental setup.

ELF-EMFs were generated in a device constructed by winding 150 turns of insulated soft copper wire with a diameter of 1.5 mm. Coils were placed vertically, facing one another (Figure 1). The magnetic flux density at the center of the coils was measured with an electromagnetic field radiation tester (EMF-827; Taiwan, China) and was adjusted by varying the coil current. Magnetic field measurements showed that under experimental conditionals, the magnetic field exposure system produced a stable flux density of 0.2 mT and a stable frequency of 50 Hz, with negligible vibration. Temperature was continuously monitored by Therm 2283–2 thermometers (Taylor Precision Instruments, Oak Brook, IL). None of the experimental conditions produced any measureable temperature increase in the cell culture medium. The exposure system was put in a humidified incubator at 37 °C, which contained 5% CO_2_. Exposed cells were placed inside the coils with a sinusoidal 50 Hz electromagnetic field at 0.2 mT for 24 h. Control cells (not exposed to ELF-EMFs) were cultured in another incubator without power coils.

RPE cells (5×10^5^ cells cultured in 25-mm^2^ plastic bottles) cultured with DMEM without FBS were exposed to ELF-EMFs (0.2 mT) for 24 h. The supernatant medium of RPE was then collected and condensed by an ultra filter device (Millipore, Billerica, MA) by spinning the device at 4,000 × *g* for 25 min. The ultrafiltrate protein concentration was measured using the Bradford Protein Assay kit (Beyotime, Shanghai, China).

### Cell proliferation assay

The proliferation rate of HFSFs treated with or without RPE supernatant medium while exposed to ELF-EMFs was evaluated with the cell counting kit-8 assay (Dojin Laboratories, Kumamoto, Japan). For the cell proliferation assay, 100 μl of passage HFSFs (2,000, 4,000, 6,000, 8,000, and 10,000 cells/well) were seeded into 96-well plates containing DMEM with 10% FBS. Cells were allowed to attach to the substratum for 12 h. The following day, the medium was replaced with fresh medium and the cells exposed to ELF-EMFs (0.2 mT, 50 Hz) were cultured in different humidified incubators (37 °C, containing 5% CO_2_) for 24 h. Control cultures were kept in the same conditions as the exposed ones without field application. In a separate 96-well plate, HFSFs were seeded at an initial density of 6,000 cells/well at various concentrations of RPE supernatant (0.0075, 0.015, 0.03, 0.06, and 0.12 mg/ml) or the control condensed medium. The cultures were exposed to ELF-EMFs (0.2 mT, 50 Hz) at 37 °C with 5% CO_2_ for 24 h. Control cultures were kept in the same conditions as the exposed ones without field application.The solution of 2-(2-methoxy-4-nitrophenyl)-3-(4-nitrophenyl)-5- (2,4-disulfophenyl)-2H-tetrazolium and 1-methoxyphenazine methosulfate (Dojindo, Japan) was then added to each well, and cells were incubated for another 2 h. Finally absorbance was measured at 450 nm using a microplate reader.

### Indirect immunofluorescence

Fibroblasts (1×10^5^ cells) were grown on coverslips in six-well plates (Corning Ltd) to 50%–60% confluence. The cells were exposed to ELF-EMFs (50 Hz, 0.2 mT) produced by the coils and treated with RPE supernatant medium (0.015 mg/ml) for 24 h. Then, the cells were then washed three times with phosphate buffered saline (0.01 M PBS: KH_2_PO_4_, 0.20 g; Na_2_HPO_4_, 1.56 g; NaCl, 8.0 g; KCl 0.20 g), covered with 10% normal goat serum diluted in PBS, and incubated for 20 min at 37 °C. The slides were incubated at 4 °C overnight with primary antibodies (anti-collagen type I diluted to 1:500 in PBS, anti-MMP-2 diluted to 1:100 in PBS; Abcam, Cambridge, UK). Cells incubated in PBS without primary antibodies were used as a negative control group. The antibody-treated and negative-controlled sample slides were washed with PBS and exposed to DyLight 488-conjugated anti-mouse immunoglobulin G (IgG) antibodies (Invitrogen Corp, Carlsbad, CA) at 37 °C for 60 min. The slides were washed three times with PBS, and cell nuclei were stained with 4', 6-diamidino-2-phenylindole (Invitrogen). Immunofluorescence images were taken using an inverted fluorescent microscope (Leica, Wetzlar, Germany).

### Quantitative real-time PCR analysis

HFSFs and RPE cells were seeded in 25-mm^2^ plastic bottles at 5×10^5^ cells and cultured for 12 h. HFSFs cultures treated with RPE supernatant medium (0.015 mg/ml) were exposed to ELF-EMFs (50 Hz, 0.2 mT) produced by the coils. After exposure for 24 h, cells were harvested for total RNA extraction by using Trizol Reagent (Invitrogen). cDNAs were synthesized with 4 µg of total RNA, according to the manufacturer’s protocol for the Reverse Transcription kit (Fermentas, Japan). Based on the sequences reported in the GenBank database (Table 1), *COL1A1*, *MMP-2*, *TGF-β2*, *FGF-2*, and glyceraldehyde-3-phosphate dehydrogenase (*GAPDH*) primers were designed, selected, and ordered using Primer-Premier 5 (Premier Biosoft Interpairs, Palo Alto, CA), BLAST, and Sangon (China), respectively. Quantitative real-time PCR was performed with an ABI7500 Fast Real-Time PCR System (Applied Biosystems, Foster City, CA). A typical reaction was performed in 25 µl, consisting of 2 µl cDNA, 12.5 µl 2X SYBR Green PCR buffer, and primer pairs (final 10 pmol each). The PCR temperature cycle was performed for 2 min at 95.0 °C, followed by 40 cycles with primer annealing for 30 s at the temperatures indicated in Table 1, and extension for 30 s at 72.0 °C. The change in threshold cycle (∆CT) was calculated by subtracting the average CT of *GAPDH* mRNA from the average CT of the target genes. All experiments were performed in triplicate. The comparative quantification values were obtained from the CT number at which the increase in signal was associated with an exponential growth of PCR products.

**Table 1:** Accession number of genes in the nucleotide sequence database (**NCBI**), sequences of used primer pairs and temperatures.

**Gene**	**Primer sequences**		**GenBank numbers**	**Tm(°C)**
*COL1A1*	F: 5′ GTGTTGTGCGATGACG 3′	R: 5′ TCGGTGGGTGACTCTG 3′	NM_000088.3	57
*MMP-2*	F: 5′ GGAAAAGATTGATGCG 3′	R: 5′ GGTGCTGGCTGAGTAG 3′	NM_004530.4	59
*TGFβ2*	F: 5′ ATCTGGTCACGGTCGC 3′	R: 5′ GTCCCTGGTGCTGTTG 3′	NM_003238.2	58
*FGF-2*	F: 5′ CGTTACCTGGCTATGA 3′	R: 5′ CAACTGGTGTATTTCCT 3′	NM_002006.4	53
*GAPDH*	F: 5′ CTCCTCCACCTTTGACGC 3′	R: 5′ CCACCACCCTGTTGCTGT 3′	NM_002046.3	60

### Western blot analysis

After exposure to ELF-EMFs (50 Hz, 0.2 mT) and treatment with or without RPE supernatant medium (0.015 mg/ml) or not, total cellular protein extract from HFSFs was obtained by lysing the cells in an ice-cold radioimmunoprecipitation assay buffer containing proteinase inhibitors (50 mM Tris–HCl, pH 8.0; 150 mM NaCl; 1% NP-40; 4 °C) and phosphatase inhibitors (R&D Systems). After centrifugation at 12,000 × *g* (30 min; 4 °C), the supernatants were collected and protein concentration measured using the bicinchoninic acid (BCA) Protein Assay kit (Beyotime). Samples containing 50 µg of protein were subjected to sodium dodecyl sulfate polyacrylamide gel electrophoresis (SDS-PAGE) polyacrylamide gel electrophoresis, using 10% and 12% polyacrylamide gel (30% acrylamide-bisacrylamide; 1.5 M Tris–HCl, pH 8.8; 10% SDS; 10% ammonium persulfate; TEMED), and then transferred onto polyvinylidene difluoride membranes (Bio-Rad Laboratories, Hercules, CA). The membrane was blocked in 5% Tris buffered saline (10 mM Tris-HCl, pH 8.0; 150 mM NaCl; 0.5% Tween-20; 5% fat-free dry milk) for 1 h at room temperature. It was then probed overnight at 4 °C with speciﬁc primary antibodies against collagen type I (1:5,000; Abcam), MMP-2 (1:1,000; Abcam), phosphor-ERK1/2, phosphor-p38, ERK1/2, p38, and β-actin (1:1,000; Cell Signaling Technology) and incubated in blocking buffer with ß-actin as an internal control. Immunoblots were then washed and incubated with a horseradish peroxidase (HRP)-conjugated anti-rabbit goat IgG HRP (1:1,000; Abmart, Shanghai, China). Membranes were developed with the ECL kit (Pierce, Thermo Scientific), according to the manufacturer’s protocol, acquired with a ChemiDoc XR system (Bio-Rad Laboratories). The relative level of protein expression was expressed as the density ratio of the protein compared to β-actin in the same sample.

### Data analysis

Statistical analysis was performed with SPSS 16.0 Statistical Software (SPSS, Inc., Chicago, IL). Data were expressed as the mean±standard deviation (SD) of at least three separate repeated experiments. The differences between exposed and untreated cells were tested using one-way analysis of variance or Dunnett’s test. Statistical significance was accepted if p<0.05.

## Results

### Changes in HFSFs proliferation

**Figure 2:**
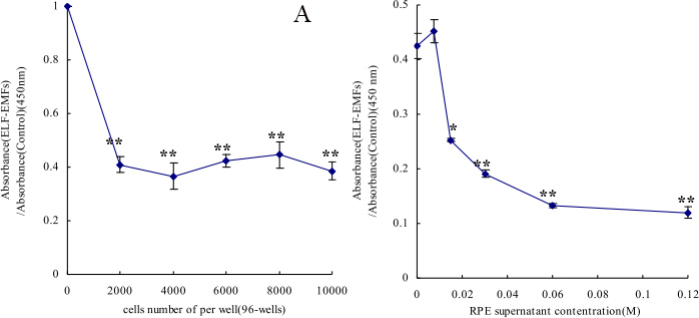
Electromagnetic fields and retinal cells can affect human scleral fibroblast cells’ proliferation. **A:** The cells cultured at initiated number of 0, 2000, 4000, 6000, 8000 and 10000 cells/well, after exposure to ELF-EMFs. The absorbance of live cells was measured by multiscan spectrum. **B:** The cells cultured at initiated number of 6000 cells/well. Numbers of live cells were determined using cell counting Kit-8 after cells were treated with various doses of RPE supernatant medium (0.0075, 0.015, 0.03, 0.06, 0.12 mg/ml) under ELF-EMFs exposure. The results are shown as the numbers of live cells in culture with RPE supernatant compared with control cultures (mean±SD). Asterisks indicat significant differences when compared with the appropriate control cultured; the asterisk denotes p<0.05 and the double asterisk stands for p<0.01.

There was a significant reduction in the proliferation rate of HFSF cells exposed to ELF-EMFs compared with control cultures (t=–3.26, p<0.05, Figure 2A). The inclusion of RPE supernatant medium resulted in a significantly reduced proliferation rate of HFSFs compared to that of the cells exposed to ELF-EMFs alone (t=–7.32, p<0.05, Figure 2B).

### Electromagnetic fields and retinal cells can affect human scleral fibroblast cells COL1A1 and MMP-2 expression

**Figure 3:**
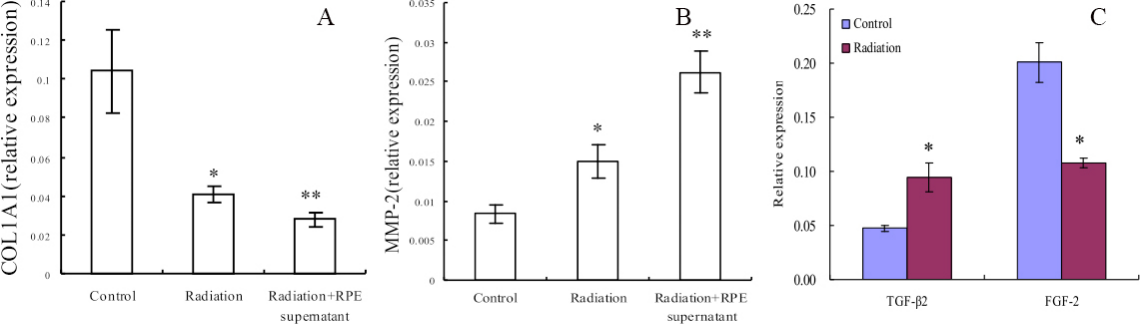
Electromagnetic fields can change *COL1A1*, *MMP-2*, *TGFβ2,* and *FGF2* mRNA expression. *COL1A1* and *MMP-2* mRNA levels were measured with real-time PCR analysis after cells were treated with ELF-EMFs with or without RPE supernatant medium. mRNA abundance is expressed as cDNA copy number relative to copies of *GAPDH*. **A:** ELF-EMFs suppressed *COL1A1* mRNA expression compared to control cells. After treatment with RPE supernatant medium, the decrease in COL1A1 mRNA levels induced by ELF-EMF exposure was accelerated. **B:** ELF-EMFs increased *MMP-2* mRNA expression compared to control cells, and RPE supernatant medium induced ELF-EMF enhancement of *MMP-2* expression. **C:** ELF-EMFs can affect *TGFβ2* and *FGF2* mRNA expression in RPE cells. Values (mean±standard error of the mean) were expressed as relative expression levels, the asterisk denotes p<0.05 and the double asterisk stands for p<0.01.

**Figure 4:**
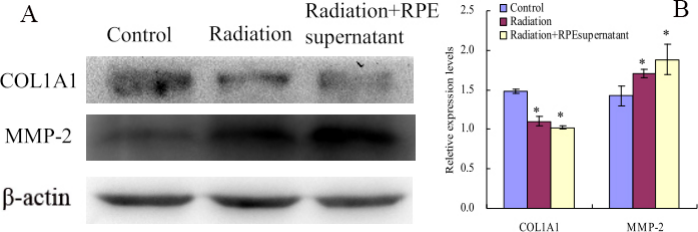
Electromagnetic fields and retinal cells can affect COL1A1 and MMP-2 protein expression in human scleral fibroblast cells. **A:** ELF-EMFs suppressed COL1A1 expression compared to control cells. Under the same radiation of ELF-EMFs, no significant difference was found in the expression of COL1A1 between the treated cells with RPE supernatant medium and the control cells. However, MMP-2 protein expression level was upregulated after treatment with ELF-EMFs and significant enhanced when treated with RPE supernatant medium under ELF-EMFs. **B:** Bar graphs show COL1A1, MMP-2 changes in protein expression (mean±SD, n=3) where density values were compared toβ-actin density. The asterisk indicates a significant difference relative to the control (p<0.05).

**Figure 5:**
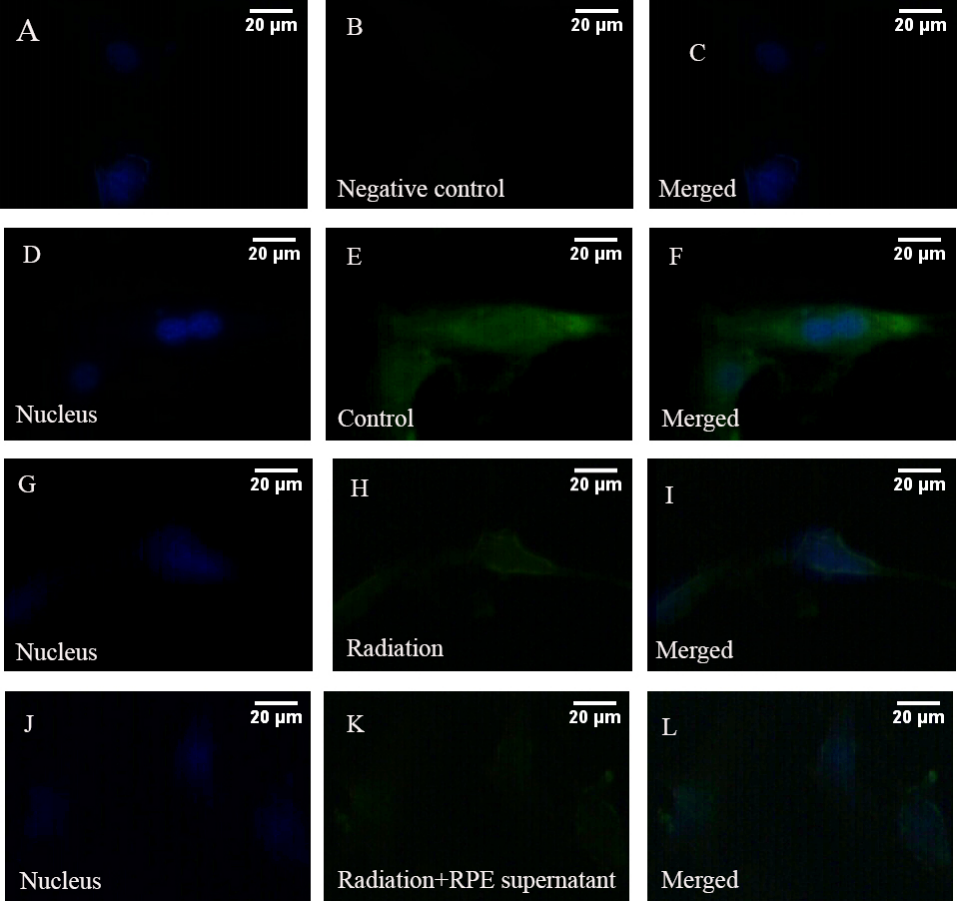
Electromagnetic fields and retinal cells can affect the expression of COL1A1. The control cells were cultured without ELF-EMFs and RPE supernatant medium. Then the cells were labeled with antibodies for COL1A1 (green). 4', 6-diamidino-2-phenylindole dyed the nucleus (blue). **A–C:** PBS was used instead of primary antibody as a negative control. **D–F:** Control cells were cultured without ELF-EMFs and RPE supernatant additive. COL1A1 was localized to the cytoplasm. **G–I:** Cells were cultured after exposure to ELF-EMFs. COL1A1 staining became weak. **J–K:** Cells were coexposed to ELF-EMFs and RPE supernatant medium. COL1A1 staining much weaker. The scale bar is 20 μm.

**Figure 6:**
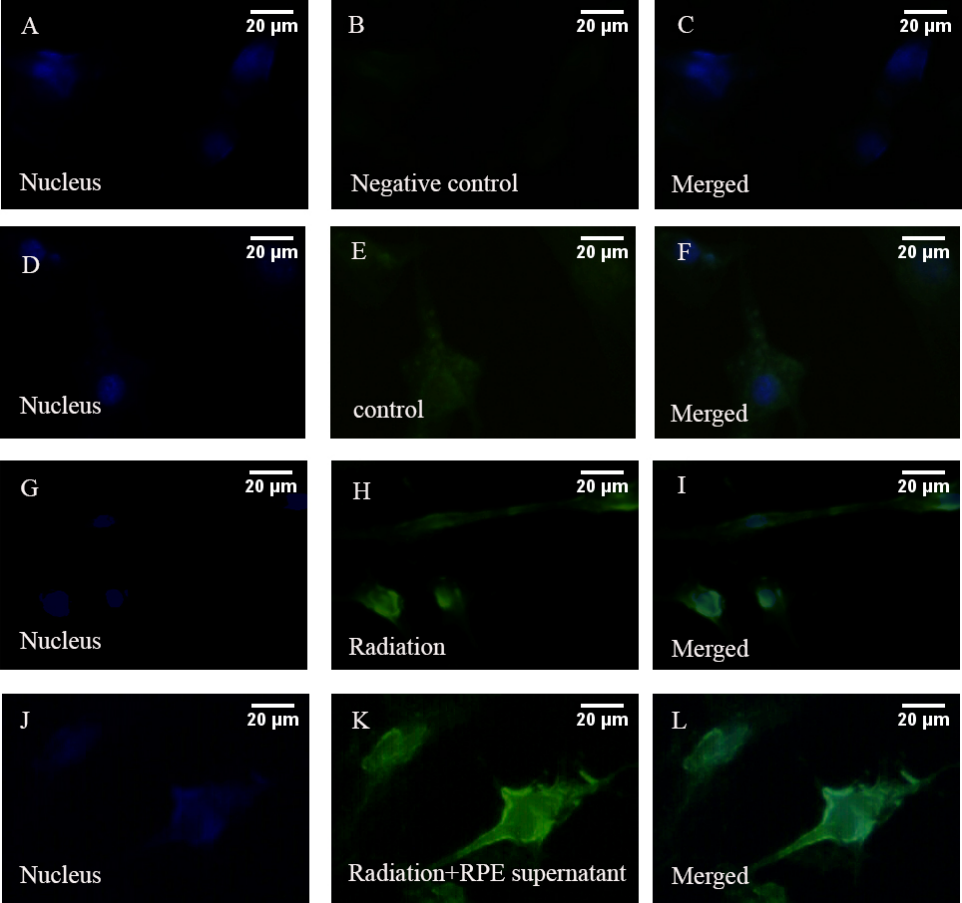
Electromagnetic fields and retinal cells can affect the expression of MMP-2. Expression of MMP-2 in HFSFs were cultured at ELF-EMFs with RPE supernatant medium or not. The control cells were cultured without ELF-EMFs and RPE supernatant additive. Then the cells were labeled with antibodies for MMP-2 (green). 4', 6-diamidino-2-phenylindole dyed the nucleus (blue). **A-C:** PBS was used instead of primary antibody as a negative control. **D-F:** Control cells were cultured without ELF-EMFs and RPE supernatant additive. **G-I:** Cells were cultured with ELF-EMFs exposure. MMP-2 showed more marked fluorescent signal than the control cells. **J-K:** Cells were coexposed to ELF-EMFs and RPE supernatant medium. MMP-2 staining became stronger than exposure to ELF-EMFs only. Scale bar=20 μm.

ELF-EMF exposure could significantly reduce the mRNA expression of *COL1A1* in HFSFs (t=–4.083, p=0.05). The effect was more obvious at the mRNA level of HFSFs exposed to ELF-EMFs with RPE supernatant additive (t=–4.987, p<0.01, Figure 3A). ELF-EMF exposure also could significantly reduce the protein expression of COL1A1 in HFSFs, but no significant difference was found for protein levels between the treated HFSFs with RPE supernatant medium (0.015 mg/ml) and the control cells exposed to ELF-EMFs (Figure 4). The mRNA level of *MMP-2*’s was upregulated in cells exposed to ELF-EMFs (t=3.919, p<0.05), and RPE supernatant medium additive could enhance the ELF-EMF effect on the expression of *MMP-2* (t=8.828, p<0.05, Figure 3B). The increased MMP-2 protein level correlated with the increased mRNA level (Figure 4). The expression of COL1A1 and MMP-2 in HFSFs was further confirmed by immunofluorescence (Figure 5, Figure 6).

### Electromagnetic fields can affect *TGFβ2*, *FGF-2* mRNA expression in retinal pigment epitheliums

Relative *TGF-β2* and *FGF-2* mRNA levels were markedly changed in RPE cells after exposure to ELF-EMFs. Significantly increased *TGF-β2* mRNA expression (t=4.45, p<0.05) and decreased FGF-2 mRNA expression (t=–5.81, p<0.05) were noted in RPE cells exposure to ELF-EMFs (Figure 3C).

### COL1A1 and MMP-2 immunofluorescence staining changes after electromagnetic fields exposure

COL1A1 was confirmed to be expressed in the cytoplasm of HFSFs. After cells were exposed to ELF-EMFs (0.2 mT, 24 h), COL1A1 staining decreased. When cells were co-exposed to ELF-EMFs and RPE supernatant medium (0.015 mg/ml) additive, COL1A1 staining was much weaker (Figure 5). However, following exposure to ELF-EMFs, the expression of MMP-2 showed more marked fluorescent signals localized in the cytoplasm of treated cells than in that of untreated cells. When cells were co-exposed to ELF-EMFs and RPE supernatant medium (0.015 mg/ml), MMP-2 staining appeared stronger than exposure to ELF-EMFs alone (Figure 6).

### Potential signal pathway of electromagnetic fields and retinal cells effects on human scleral fibroblast cells

**Figure 7:**
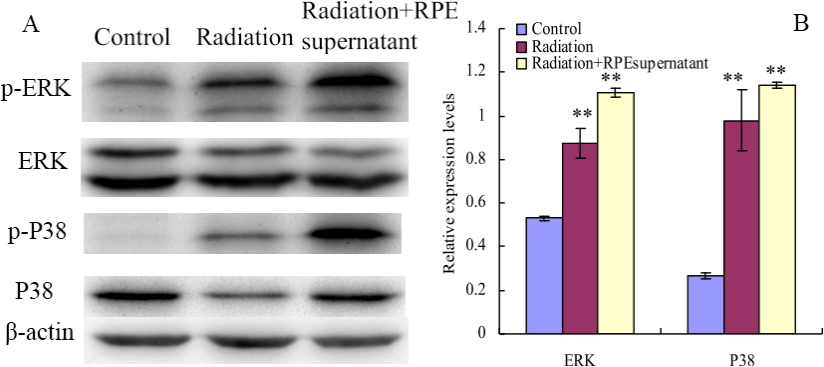
Electromagnetic fields and retinal cells can affect the phosphorylation of ERK1/2, P38, Akt and JNK in human scleral fibroblast cells. Effect of exposure to ELF-EMFs on the phosphorylation of ERK1/2, P38, Akt and JNK in HFSFs treated with RPE supernatant or not. **A**: Western blot analysis was performed using p-ERK1/2, p-p38, ERK1/2, p38, and β-actin antibodies, respectively. **B**: Densitometric quantification of Phosphorylation levels were obtained by optical density (O.D.) of protein bands normalized with O.D. of β-actin band. Values (mean±SD, n=3) were expressed as relative expression levels. A quantitative analysis was performed by comparison with the untreated control. The double asterisk stands for p<0.01.

Western blots showed that the expression of p-p38 and p-ERK1/2 protein levels were significantly increased (Figure 7) after cells were exposed to ELF-EMFs (0.2 mT, 24 h). The expression level was higher when exposed to ELF-EMFs with RPE supernatant medium (0.015 mg/ml) additive (Figure 7).

## Discussion

Collagen synthesis in keloid fibroblasts has been shown to be inhibited when exposed to EMFs with 60 Hz for 10 days [29]. Collagen synthesis could be decreased in guinea pig’s skin after exposure to 1 mT ELF-EMFs [30], and the expression of collagen I mRNA could be downregulated following EMF stimulation [31]. As an important component of sclera, type I collagen is involved in pathological scleral matrix remodeling. Our results demonstrated that exposure to ELF-EMFs downregulated mRNA and protein expression levels of COL1A1 in HFSFs. Moreover, under the same ELF-EMF exposure conditions, there was a significant difference in mRNA levels between cells treated with RPE supernatant medium and the control cells (Figure 3A, Figure 4A, Figure 5). These results indicate that ELF-EMFs can suppress collagen synthesis in the sclera and might induce sclera remodeling, which is a potential risk factor for the eye’s elongation.

MMPs could affect scleral ECM during scleral remodeling by inhibiting collagen type I synthesis [32]. In this study, we found that, compared with the control cells, both the protein and mRNA expression levels of MMP-2 were upregulated with exposure to ELF-EMFs, and MMP-2 expression was further enhanced with the inclusion of RPE supernatant medium (Figure 3B, Figure 4B, Figure 6). These data indicate that ELF-EMFs might act to enhance the initiation process of scleral remodeling by increasing the expression of MMP-2.

RPE cells can synthesize and secrete cytokines and enzymes to regulate the proliferation of scleral fibroblasts, regulate the production of collagen, and further affect the development of myopia [33]. We therefore detected the mRNA expression levels of TGF-β2 and FGF-2 in RPE cells and found the mRNA expression of TGF-β2 was enhanced but that of FGF-2 decreased after exposure to an EMF for 24 h. TGF-β could induce MMP production from fibroblasts by interfering with Smad and MAPK pathways in vitro [34]. TGF-β also plays an inhibitory role of human scleral fibroblasts attachment to collagen type I in vitro and modulates scleral cell–matrix interactions in vivo [35]. In this study, FGF-2 mRNA was decreased in RPE cells after 24 h of exposure to an EMF. These results suggest that ELF-EMFs may regulate sclera changes though regulating cytokines of RPE.

Scleral fibroblasts are involved in scleral remodeling and play an important role in maintaining eye size [8]. Our results showed that following exposure to electromagnetic field, HFSFs proliferation was significantly decreased, which implied that ELF-EMFs suppress collagen synthesis might be mediated by inhibit HFSFs proliferation, and retinal is the source of ocular growth-regulating signals [12]. Therefore, the decreased proliferation of HFSFs by EMFs might also via activate some signal pathways in RPE cells.

It has been reported that MAPKs can be activated in response to a variety of environmental stresses including radiation [36]. ERK1/2 and p38 MAPK play important roles in the collagen content exposed to ELF-EMFs [37]. Inactivation of the ERK1/2 MAPK signaling pathway can increase the expression of type I collagen in fibroblasts [38]. MAPKs can also regulate the expression of MMP-2; for example, MMP-2 can be stimulated by cyclic strain in endothelial cells in vitro, in part through both p38- and ERK-dependent pathways [39]. Inhibiting the activity of ERK could downregulate the expression of MMP-2 in fibroblasts [40]. Therefore, we tried to identify whether ELF-EMFs can regulate the content change of collagen through the MAPK pathway. We found that the collagen content decreased in HFSFs in almost all experiments (Figure 3A, Figure 4A, Figure 5). In contrast, increased expressions of ERK1/2 and p38 MAPK phosphorylation were detected when the cells were exposed to ELF-EMF and RPE supernatant medium. These results suggest that the suppression of collagen expression might be associated with the activation of the ERK1/2 and p38 MAPK pathways.

In conclusion, ELF-EMFs can regulate collagen synthesis in HFSFs by regulating the MAPK pathways and can contribute to scleral remodeling. Additional studies will be required to clarify which cytokines and/or enzymes participate in regulating collagen synthesis and MMP-2 activity in RPE supernatant medium exposed to ELF-EMFs.
